# The Processing Mechanisms of Two Types of Mixed Prospective Memory

**DOI:** 10.3389/fpsyg.2021.792852

**Published:** 2021-12-16

**Authors:** Jiaqun Gan, Yunfei Guo, Enguo Wang

**Affiliations:** Faculty of Education, Henan University, Kaifeng, China

**Keywords:** mixed prospective memory, processing mechanisms, attention, dynamic multiprocess theory, cognitive load

## Abstract

Mixed prospective memory (MPM) needs to be executed when both external time and event cues appear. According to the clarity of time cues, MPM can be further divided into two types: time-point MPM and time-period MPM. There is no research on these two types of MPM. Whether existing theories of EBPM can explain its processing mechanisms is worth exploring. The current study was aimed at examining the differences in attentional allocation characteristics between these two types of MPM and EBPM under different difficult ongoing tasks. The results showed that the attention consumption of the two types of MPM groups was less than that of the EBPM group in the early and middle stages of high cognitive load, but there was no difference between the three groups in the later stage of the task. The attention distribution of time-point MPM and time-period MPM displayed dynamic changes: the time-point MPM only had attention consumption in the later stage, while the time-period MPM also existed in the early and middle stages. These results support dynamic multiprocess theory.

## Introduction

The ability to remember to fulfill an intention at a certain point in the future is referred to as prospective memory (PM; Einstein and McDaniel, 1990). According to the nature of PM cues, PM can be divided into event-based prospective memory (EBPM) and time-based prospective memory (TBPM). Event-based prospective memory needs to be implemented when there are clear cues from the outside (for example, remembering to buy bread when passing through the bakery). Time-based prospective memory needs to be performed at a specific time without clear external cues (for example, remembering to have a meeting at 3 o’clock tomorrow afternoon). However, most people’s lives are often planned and regular. Regular EBPM tasks tend to have an expected time (for example, going to the breakfast shop on duty to buy breakfast, and going from home to the breakfast shop takes about 10 min). Repeated TBPM tasks also have obvious external cues due to highly planned living arrangements (for example, a person should remind her diabetic mothers to take insulin at 11 o’clock, and, at this time, she usually picks up the child at the school gate). A large part of our lives consists of PM tasks that have both time and event cues. Such a PM task is called mixed prospective memory (MPM; Block and Zakay, 2006).

Compared with EBPM, MPM may have significantly different processing mechanisms due to the additional time cues. Currently, there are three main theories used to explain EBPM. Preparatory attentional processes and memory processes theory (PAM) suggest that once an individual effectively encodes a PM task, the individual still monitors the target cues even if the PM task has not yet appeared. Therefore, the successful execution of PM usually consumes cognitive resources (Smith and Bayen, 2006; Smith and Loft, 2014). According to the multiprocess theory, in most cases, the maintenance and retrieval of PM tasks require cognitive resources. However, in some specific cases, the successful execution of PM tasks may not involve cognitive resources. For example, under the conditions of prominent cues, focused cues, and simple tasks, the performance of PM tasks does not necessarily interfere with the ongoing task (Yuan et al., 2011; Abney et al., 2015).

Dynamic multiprocess theory is a viewpoint proposed in recent years to explain the processing mechanism of PM. It holds that individuals’ occupation of cognitive resources in the process of successfully performing PM tasks is not a simple “all-or-nothing” relationship. Instead, cognitive resources are invested selectively and dynamically according to the characteristics of the task. For example, individuals can flexibly and selectively monitor the PM task when the occurrence time or context of the PM target is predictable. Before the PM cues appear, only attention resources need to be devoted to the ongoing task; When the PM cues are about to emerge, the attention resources need to be transferred from ongoing tasks to search and monitor PM cues (Chen and Huang, 2009; Chen et al., 2010; Scullin et al., 2013).

The predictable EBPM described by dynamic attention theory is essentially like MPM. Therefore, when performing MPM, the individual’s attention process should display the characteristics of dynamic change. Some research supports this view. Chen et al. (2010) compared the PM interference effects (the phenomenon that the PM task interferes with the performance of the ongoing task) of MPM and EBPM on the ongoing task at different time stages. Under MPM conditions, the participants were informed that the PM target words appeared 10 min after the beginning of the experiment; Under EBPM conditions, the participants were not told when the PM cue appeared. The results found that EBPM had a PM interference effect at all stages, while MPM only had this interference after entering the target stages. This indicates that the participants could selectively and dynamically invest the attention resources into different task stages based on the time information during the implementation of MPM. Chen et al. (2014) further used the event-related potential technology to verify that the processing mechanism of MPM was different from EBPM, showing the characteristics of dynamic and flexible attention allocation. According to the above evidence, MPM has additional time cues compared with EBPM. When performing MPM, by estimating the length of time from the beginning of the task to the emergence of PM cues, individuals can flexibly allocate attention and improve attention efficiency.

The processing mechanism of MPM may be affected by some factors, such as the clarity of time cues. If the time cue of MPM is a definite time point, then the time information provided by MPM is relatively straightforward. Individuals can accurately predict the time when the MPM cue appears, and they can flexibly allocate attention according to the apparent time information. If the time cue of MPM is a time range, then the time information it provides is relatively vague. Individuals may need to pay more attention to searching for external cues, and the flexibility of attention may be poor.

Besides, the predictability of contextual cues will enable individuals to implement efficient planning strategies during task execution (Cook et al., 2005). When providing contextual information on the appearance of PM cues, participants will strategically restrict the attention monitoring to the context in which the PM cues were expected and potentially formulate a plan to conserve resources appropriately (Marsh et al., 2006). Although MPM does not provide contextual information, its time information also improves the planning and strategy of tasks. Therefore, according to the clarity of time cues, to examine the processing mechanism of MPM, the current study divides MPM into two types, time-point MPM and time-period MPM.

In addition, the processing mechanism of MPM may be affected by cognitive load. Cona et al. (2015) showed that the high cognitive load of the ongoing task prevents individuals from engaging in strategic monitoring, resulting in minimal involvement of the dorsal frontoparietal and cognitive control regions. Momennejad and Haynes (2013) found that, when the ongoing task load is relatively high, individuals are more likely to rely on automatic processing, which is more dependent on the temporary activation of the insula/ventral frontoparietal network. Some studies have found that the training effect of time estimation is affected by the difficulty of the background tasks. When the background task is difficult, individuals have a large deviation in time estimation performance (Taatgen et al., 2007). This performance does not improve even after short-term training (Lio et al., 2006).

Consequently, the accuracy of the time estimation is affected by the cognitive load. If the difficulty of the ongoing task increases and the accuracy of the time interval estimation is poor, will the attention allocation still show the characteristics of flexibility? In particular, under the condition of high cognitive load in the time-period MPM, will individuals still flexibly and dynamically allocate the limited capability of attention resources through the processing of time information? This research intended to focus on the attention assignment characteristics of two different types of MPM under different cognitive loads. According to the prediction of the PAM theory, multiprocess theory and dynamic multiprocess theory, if the participants had attention consumption in the whole process of performing PM task, and there was no difference between each stage, it supported the PAM theory; If the participants did not consume attention resources throughout the process, it supported the multiprocess theory; if the participants’ attention allocation showed a dynamic change, and there was no significant attention consumption when the target cue was far away (early and middle stage), but attention consumption increased when the target cue was about to appear (later stage), which supported the dynamic multiprocess theory.

## Materials and Methods

### Participants and Design

One hundred and eleven college students at Henan University (*M*_age_ = 20.1, range = 18–25) participated in the experiment. All the participants had normal or corrected vision and never participated in similar experiments. They were required to sign informed consent forms before the experiment and received a small reward when the experiment was finished. The study was approved by the Ethics Committee of Henan University.

A mixed design of 4 (groups: EBPM, time-point MPM, time-period MPM, baseline) × 2 (cognitive load: low, high) was adopted. The group was a between-subject factor, and the cognitive load was a within-subject factor. All the participants were randomly assigned four experimental conditions as follows: EBPM group (*N* = 25, *N*_male_ = 7, *M*_age_ = 21.00, *SD* = 1.29), time-point MPM group (*N* = 29, *N*_male_ = 6, *M*age = 21.00, *SD* = 1.21), time-period MPM group (*N* = 29, *N*_male_ = 9, *M*_age_ = 20.31, *SD* = 1.51), and baseline group (*N* = 28, *N*_male_ = 6, *M*_age_ = 21.07, *SD* = 1.51).

### Apparatus, Materials, and Tasks

The experiment used E-prime 2.0 to compile the program and present the experimental instructions, stimulus items, and data collection on the computer. All the materials were presented in 22-point font at the center of an 18” LED monitor. The experimental procedure was controlled by the E-Prime 2.0 software program running on Dell computers. Participants were tested individually in a sound-attenuated booth and performed the ongoing task and PM task response by pressing specific keys on the keyboard.

The experimental materials were 24 English capital letters (excluding the letters F and J), displayed on a gray background with black font. The PM cues were the letters G and R, and the ongoing task stimuli were randomly selected from the remaining 22 letters.

This experiment adopted Smith’s paradigm of prospective interference effect (Smith and Bayen, 2004). That is, four conditions were set in the experiment. The control condition was a simple ongoing task, and the other three experimental conditions were a PM task embedded in an ongoing task. To examine whether the implementation of the PM task interfered with the ongoing task, the behavior data of non-PM targets under the control and experimental conditions were compared. The ongoing task used the N-back paradigm to manipulate cognitive load (Chen et al., 2017). The 1-back task required participants to compare the current letter with the first letter preceding it (the first letter of the program did not need to be reacted, and the reaction started from the second letter). If the two letters were the same, then the participants were instructed to press the J key with the right forefinger; otherwise, they were instructed to press the F key with the left forefinger. The 2-back task was like the 1-back task except that it required participants to compare the current letter with the second letter preceding it (the first two letters of the program did not need to respond). To balance the sequential effect, half of the participants performed the 1-back task first and the 2-back task second, while the other half did the opposite. The PM task was to press the spacebar when encountering the letter G or R.

The baseline group only performed the ongoing task. The EBPM group did not inform the participants when the PM cue would appear, the time-point MPM group told the participants that the PM cue would appear at exactly 1 min, and the time-period MPM group told the participants that the PM cue would appear after 1 min. There were four blocks in the procedure, and the participants would take a break every 69 s. After each break, the letter comparison task started from the beginning, and the timing of the procedure started at 0:00.

### Procedure

The procedure started with instructions of the ongoing task. After participants correctly understood the instruction, they entered the practice phase (In order to ensure that the participants could perform the ongoing task quickly and accurately, the practice phase included 50 ongoing task trials without PM task). At the beginning of each ongoing task, a fixation (+) appeared in the center of the screen for 500 milliseconds (ms). Then, a capital letter appeared in the same position for up to 2,000 ms and disappeared when participants responded. Next, a blank screen would appear as a buffer for 500 ms, and then, the trial ended. After the practice phase, the formal experiment that consisted of four blocks in all the groups was carried out. There were 23 trials of each block, of which four PM cues were inserted (two for each of G and R). The EBPM and time-point MPM groups inserted PM cues once at the position of the 20th trial in each block. The time-period MPM group inserted a PM cue at the position after the 19th trial of each block (appearing in the positions of the 23rd, 20th, 22nd, and 21st trials of the four blocks). At the end of the experiment, all the participants were asked whether they remembered the PM task.

## Results

### Prospective Memory Performance

**Figure 3:**
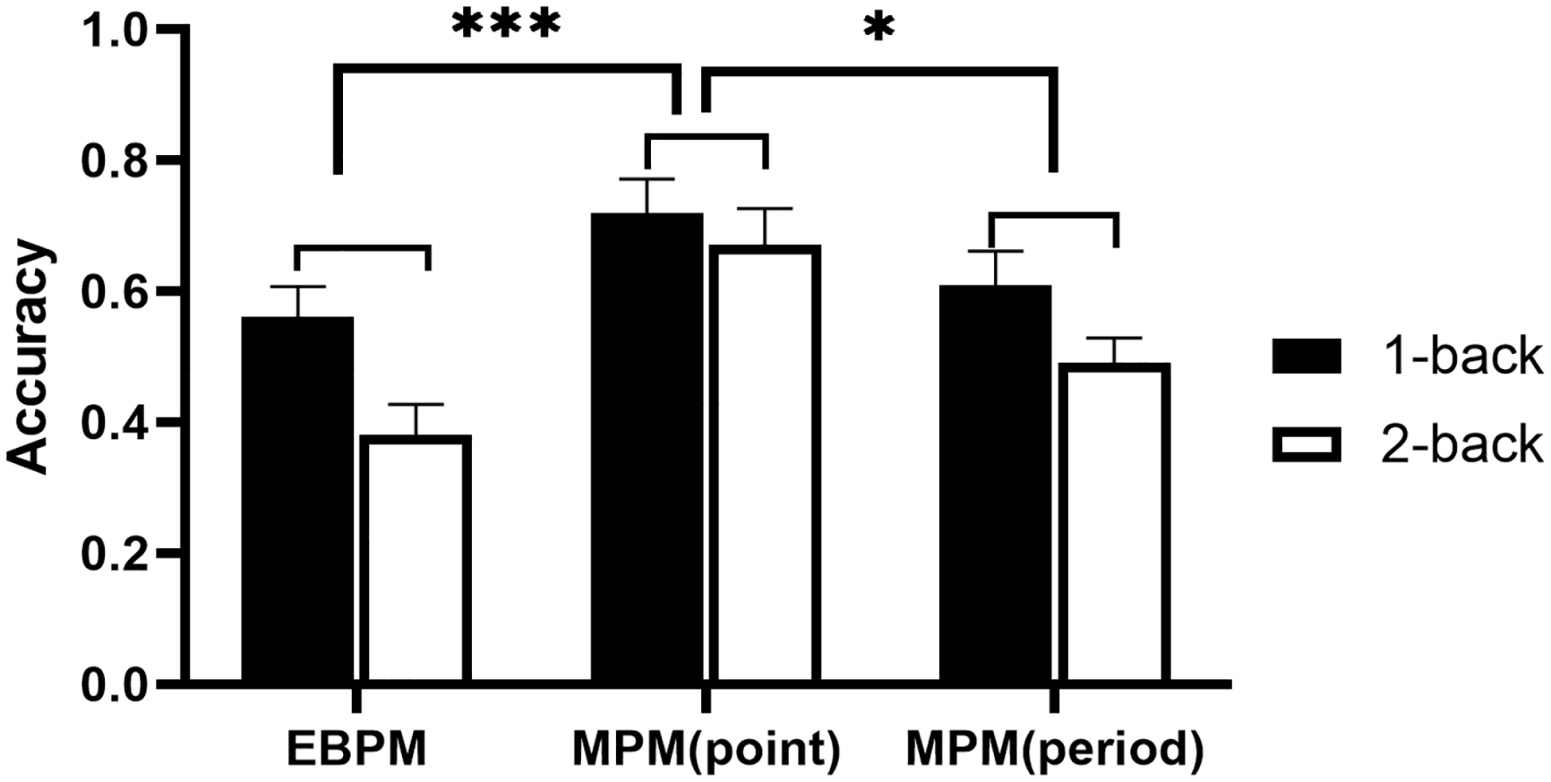
The accuracy of different types of prospective memory. *p < 0.05; ***p < 0.01.

A repeated measure ANOVA of 3 (groups: EBPM, time-point MPM, time-period MPM) × 2 (cognitive load: low, high) was conducted on the accuracy of PM. The results showed that the main effect of the cognitive load was significant, *F*(1, 80) = 9.89, *p* < 0.01, $\eta_{p}^{2}$ = 0.11, and the accuracy of PM under low cognitive load was better than that under high cognitive load; the main effect of the group was significant, *F*(2, 80) = 7.72, *p* < 0.001, $\eta_{p}^{2}$ = 0.16, and the PM accuracy of the EBPM group and the time-period MPM group were lower than that of the time-point MPM group, *ps* < 0.05. The rest of the results was not significant (only statistically significant results are presented here due to the complexity of this study). (The accuracy of prospective memory is displayed in Figure 3).

The results of repeated measures ANOVA of the response time of PM revealed that the main effect of the cognitive load was significant, *F*(1, 80) = 46.47, *p* < 0.001, $\eta_{p}^{2}$ = 0.37; the response time under low cognitive load was faster than that under high cognitive load.

### Ongoing Tasks Performance

The performance of the ongoing task was an analysis of non-target trials after the PM cues were excluded. The accuracy of the ongoing tasks was analyzed by repeated measures ANOVA of 4 (groups: EBPM, time-point MPM, time-period MPM, baseline) × 2 (cognitive load: low, high). The results showed that the main effect of the group was significant, *F*(3, 107) = 23.66, *p* < 0.001, $\eta_{p}^{2}$ = 0.40, and the time-point MPM group was higher than that in the time-period MPM and EBPM groups, while the baseline group was higher than that in all other groups, *ps* < 0.05; the main effect of the cognitive load was significant, *F*(1, 107) = 378.58, *p* < 0.001, $\eta_{p}^{2}$ = 0.37, and the accuracy under low cognitive load was higher than that under high cognitive load; the interaction between groups and cognitive load was significant, *F*(1, 107) = 22.85, *p* < 0.001, $\eta_{p}^{2}$ = 0.39; and under the condition of high cognitive load, the time-point MPM group was higher than the time-period MPM and EBPM groups, while the baseline group was higher than all other groups.

The results of repeated measures ANOVA of the response time of ongoing tasks showed that the main effect of the cognitive load was significant, *F*(1, 107) = 285.36, *p* < 0.001, $\eta_{p}^{2}$ = 0.73, and the response time under low cognitive load was faster than high cognition load; the main effect of groups was significant, *F*(1, 107) = 24.24, *p* < 0.001, $\eta_{p}^{2}$ = 0.41, and the EBPM group was slower than all other groups.

### Performance of Ongoing Tasks at Different Task Stages

To examine the interference effect of the PM tasks on the ongoing tasks and the participant’s attention allocation strategy during the whole task process, we adopted the method of Chen et al. (2010) and divided the 24 trials (23 non-target trials and 1 target trial) in each block into three stages (the early stage, the middle stage, and the later stage) in average with 8 trials as units for analysis. The accuracy and response time of ongoing tasks in different stages were analyzed for 4 (groups: EBPM, time-point MPM, time-period MPM, baseline) × 2 (cognitive load: low, high) × 3 (stages: early, middle, later) repeated measures ANOVA.

The results of the accuracy of ongoing tasks at different stages (see Table 3) showed that the main effect of the group was significant, *F*(3, 107) = 23.52, *p* < 0.001, $\eta_{p}^{2}$ = 0.40, and the time-point MPM group was higher than the time-period MPM group and EBPM group, while the baseline group was higher than all other groups, *ps* < 0.01; the main effect of the cognitive load was significant, *F*(1, 107) = 379.27, *p* < 0.001, $\eta_{p}^{2}$ = 0.78, and the low cognitive load was higher than high cognitive load, *p* < 0.001; and the main effect of the stages was significant, *F*(1, 107) = 47.25, *p* < 0.001, $\eta_{p}^{2}$ = 0.31, and the accuracy in the early and middle stage was better than that in the later stage.

**Table 3:** The accuracy of ongoing tasks at different stages (*M* ± *SD*).

	Low cognitive load			High cognitive load		
	Early stage	Middle stage	Later stage	Early stage	middle stage	Later stage
EBPM	0.97(0.04)	0.96(0.03)	0.95(0.04)	0.83(0.06)	0.80(0.06)	0.81(0.05)
Time-point MPM	0.96(0.05)	0.96(0.04)	0.94(0.05)	0.91(0.03)	0.92(0.04)	0.81(0.08)
Time-period MPM	0.97(0.04)	0.97(0.04)	0.93(0.06)	0.87(0.06)	0.85(0.05)	0.80(0.07)
Baseline	0.96(0.04)	0.96(0.04)	0.96(0.04)	0.93(0.04)	0.92(0.04)	0.91(0.05)

The interaction between stages and groups was significant, *F*(3, 107) = 9.59, *p* < 0.001, $\eta_{p}^{2}$ = 0.21. In the time-point MPM group and time-period MPM group, the accuracy in the early and middle stages was higher than that in the later stage; the interaction between cognitive load and groups was significant, *F*(3, 107) = 22.93, *p* < 0.001, $\eta_{p}^{2}$ = 0.39. Under the condition of high cognitive load, the accuracy of the time-point MPM group was higher than that of the EBPM group, while that of the baseline group was higher than that of all other groups. The interaction between stages and cognitive load was significant, *F*(3, 107) = 13.28, *p* < 0.001, $\eta_{p}^{2}$ = 0.11. Under different cognitive loads, the accuracy in the early and middle stages was higher than that in the later stage.

**Figure 1:**
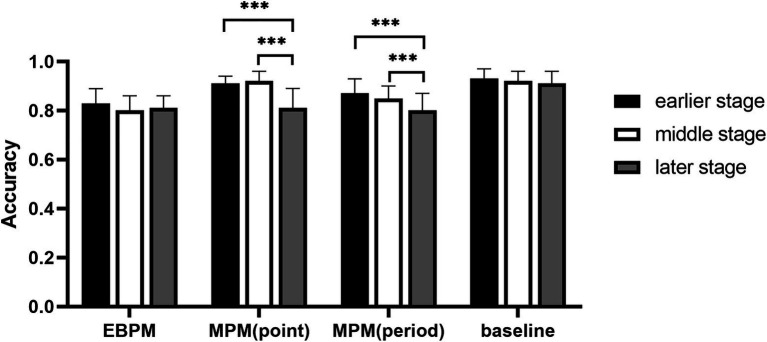
The accuracy of ongoing tasks in different groups at different stages under high cognitive load. ***p < 0.01.

**Figure 2:**
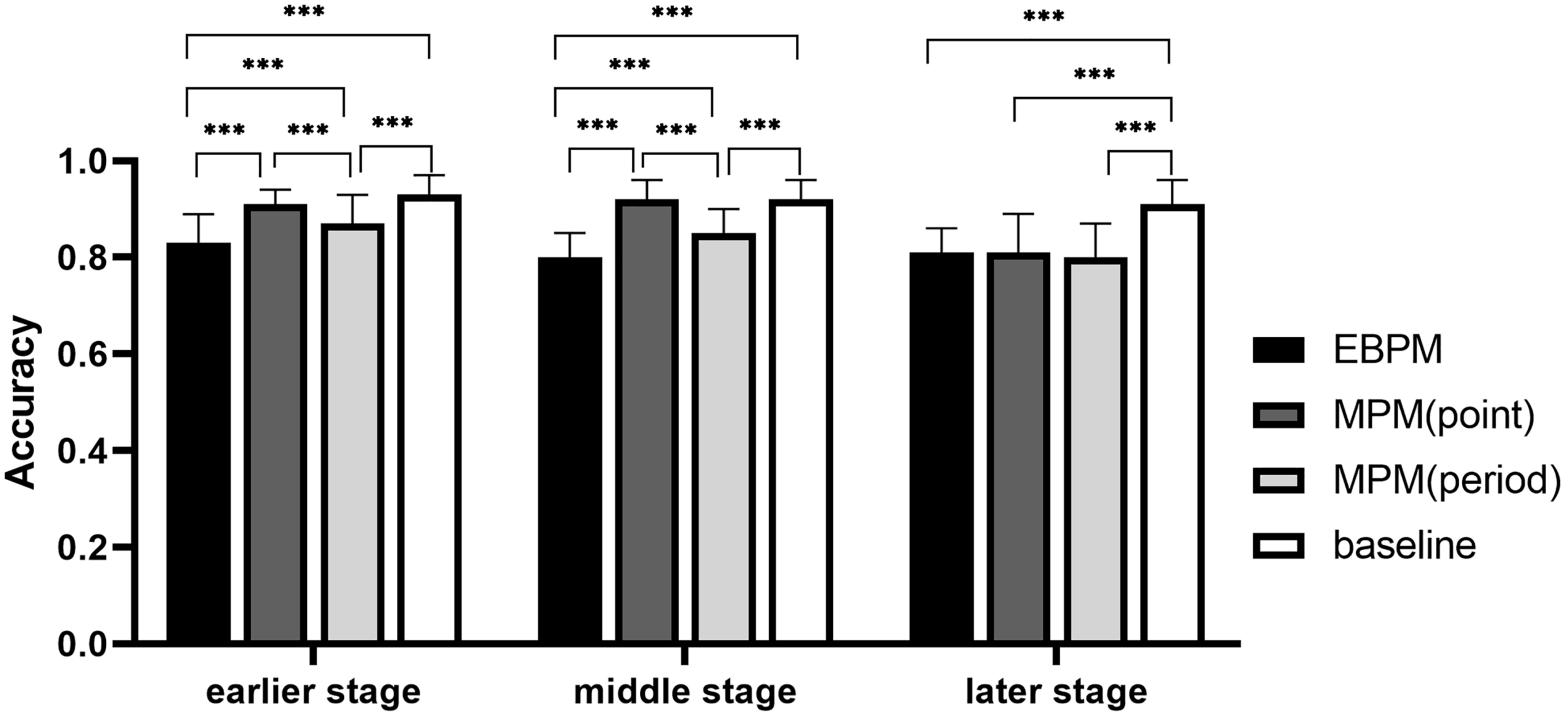
The accuracy of ongoing tasks in different groups at different stages under high cognitive load. ***p < 0.01.

The interaction between groups, cognitive load, and stages was significant, *F*(3, 107) = 3.62, *p* < 0.05, $\eta_{p}^{2}$ = 0.09. In the early and middle stages under high cognitive load, the accuracy of the time-point MPM group and the baseline group was higher than that of the time-period MPM group, while the EBPM group was lower than all other groups; in the later stage of the high cognitive load, the accuracy of the baseline group was better than that of other groups, but there was no difference between other groups (see Figure 1). In the time-period MPM group under the low cognitive load, the accuracy in the early and middle stages was higher than that in the later stage; in the time-point MPM group and time-period MPM group under the high cognitive load, the accuracy in the early and middle stages was also higher than that in the later stage (see Figure 2).

The results of the response time of the ongoing tasks at different stages (see Table 4) showed that the main effect of stages was significant, *F*(1, 107) = 42.03, *p* < 0.001, $\eta_{p}^{2}$ = 0.28, and the response time in the early stage was faster than that in the later stage; the main effect of cognitive load was significant, *F*(1, 107) = 285.36, *p* < 0.001, $\eta_{p}^{2}$ = 0.73, and the response time under low cognitive load was faster than that under high cognitive load; and the interaction between groups and stages was significant, *F*(3, 107) = 3.42, *p* < 0.05, $\eta_{p}^{2}$ = 0.09. In the time-point MPM group, the response time in the early and middle stages was faster than in the later stage; in the time-period MPM group, the response time in the early stage was faster than that in the middle and later stages. The others were not significant. (The performance of ongoing tasks and prospective memory tasks are displayed in Table 1 and Table 2).

**Table 1:** The accuracy of ongoing task and prospective memory (*M* ± *SD*).

	Ongoing tasks		Prospective memory	
	Low cognitive load	High cognitive load	Low cognitive load	High cognitive load
EBPM	0.96(0.03)	0.81(0.03)	0.56(0.26)	0.38(0.26)
Time-point MPM	0.95(0.04)	0.88(0.04)	0.72(0.28)	0.67(0.31)
Time-period MPM	0.95(0.03)	0.84(0.04)	0.61(0.28)	0.49(0.21)
Baseline	0.96(0.03)	0.92(0.03)	–	–

**Table 2:** The response time of ongoing tasks and prospective memory (*M* ± *SD*).

	Ongoing tasks		Prospective memory	
	Low cognitive load	High cognitive load	Low cognitive load	High cognitive load
EBPM	810(60)	1184(215)	905(142)	1255(324)
Time-point MPM	697(61)	977(132)	863(226)	1065(267)
Time-period MPM	717(64)	978(180)	895(117)	1129(353)
Baseline	682(55)	951(135)	–	–

**Table 4:** The response time of ongoing task in different stages (*M* ± *SD*).

	Low cognitive load			High cognitive load		
	Early stage	Middle stage	Later stage	Early stage	Middle stage	Later stage
EBPM	787(92)	815(78)	1198(254)	1158(220)	1196(222)	1198(254)
Time-point MPM	677(93)	703(70)	1042(147)	926(150)	963(156)	1042(147)
Time-period MPM	671(93)	729(103)	1016(166)	930(202)	989(196)	1016(166)
Baseline	676(74)	678(74)	967(131)	946(155)	941(163)	967(131)

## Discussion

By manipulating the clarity of PM cues and the difficulty of ongoing tasks, this study examined whether there were differences between the processing mechanism of MPM and EBPM along with the attention characteristics of two different types of MPM during the execution of PM tasks. The results showed that the performance of the time-point MPM was better than that of the time-period MPM and EBPM. In terms of the processing mechanism, the attention consumption of the time-point and time-period both displayed dynamic changes. In the early and middle stages of high cognitive load, the attention consumption of the two types of MPM groups was less than that of the EBPM group, and there was no difference between the two MPM groups and the EBPM group in the later stage. However, in the early and middle stages of high cognitive load, the processing mechanism between the time-point MPM and time-period MPM was also different. There was no evidence of attention consumption in the early and middle stages of the time-point MPM, while time-period MPM existed. These results provide evidence for dynamic multiprocess theory.

Compared with EBPM, MPM has additional time information, and individuals can rely on the time information to form better time expectations, thus having better PM performance (Chen et al., 2010). The first purpose of this study was to verify whether MPM with additional time cues would perform better than EBPM by comparing the performance of EBPM and MPM. The results showed that the performance of the time-point MPM was significantly better than that of the EBPM, but there was no difference between the time-period MPM and EBPM. These findings partly support the research of Chen et al. We did not find any difference between the performance of the time-period MPM and the EBPM. The reason for this may be that the time-period MPM cues were more ambiguous than those in the time-point MPM. In uncertain situations, individuals consume more attention (Rose et al., 2010) and have a more significant cognitive bias (Mussweiler and Strack, 2000). Therefore, compared with the time-point, the time information provided by the time-period MPM contributed less to the PM.

The second purpose of this study was to explore the processing mechanism of MPM. Numerous previous studies have found that, in most cases, the successful implementation of the EBPM tasks always requires much attention resources (McDaniel and Einstein, 2000; Smith, 2003). The results of our study showed that the performance of the EBPM’s ongoing task was significantly better than that of all other groups. There was no difference between the ongoing task performance of each stage, indicating that the EBPM task had significant attention consumption in each stage, which verified the results from previous studies (Chen et al., 2010). Compared with EBPM, MPM is predictable because of its additional time information. Dynamic multiprocess theory holds that individuals can monitor the PM task flexibly and selectively when the time or context of the PM task can be predicted. Before the PM cue appears, cognitive resources only need to be invested in performing the ongoing task. Near the emergence of the PM cue, individuals will invest many cognitive resources to search and monitor the PM cue, and thus, their attention consumption will show dynamic changes (Chen et al., 2010; Scullin et al., 2013; Moyes et al., 2019). Current PM research in a laboratory usually adopts the dual-task paradigm. The PM interference effect refers to the phenomenon that the retention and execution of PM intention compete for the cognitive resources of the ongoing task under the dual-task paradigm. Therefore, the performance of the ongoing task is usually used to reflect the individual attentional changes in the PM tasks (McNerney and West, 2007; Boag et al., 2019). This study focused on the performance of ongoing tasks.

First, we compared the performance differences between the MPM and the EBPM groups to explore how the time cues caused attention changes in MPM tasks. This study found that the response speed of the time-point MPM and the time-period MPM was faster than that of the EBPM in overall ongoing task performance, which showed that additional time information reduced the individual’s attention consumption. We further compared the performance of the ongoing tasks in different stages and found that the performance of the time-point MPM and time-period MPM groups was better than that in the EBPM group only in the early and middle stages under high cognitive load, suggesting that additional time cues reduced the attention consumption of MPM groups in the early and middle stages and made the individual attention distribution display the characteristics of dynamic changes.

Neither PAM theory nor multiprocess theory can explain dynamic characteristics of attention allocation to PM targets. According to PAM theory (Smith, 2003), before the PM target appears, there is a continuous preparatory attention processing to monitor possible targets in the environment and produce a relatively stable PM interference effect on background tasks. Multiprocess theory holds that the input of attention resources in the execution of PM tasks is “all-or-nothing,” and EBPM relies either on controlled processing or automatic processing (Einstein et al., 2005), which still cannot explain the phenomenon that attention resources are selectively invested in different time stages during the MPM task. The current research results were consistent with the prediction of dynamic multiprocess theory. Individuals can selectively and dynamically invest attention resources according to the characteristics of the tasks. Because the time or context of PM cues is predictable in MPM, the individual monitoring of PM tasks can be flexible and selective. In the non-target stage, when the PM cue does not appear, attention resources are only needed to perform the ongoing task. In the target stage, when the PM cue appears, attention resources are transferred from the ongoing task to the PM task.

Secondly, to what extent did the MPM group reduce attention consumption in the early and middle stages? Since the baseline group did not perform the PM task in the whole task, the performance of the ongoing task between the MPM group and the baseline group could be compared to explore whether the attention consumption of MPM in different stages reached the level of automatic processing. We found that there was no difference in the accuracy of ongoing tasks between the time-point MPM group and the baseline group in the early and middle stages under high cognitive load, while the difference between the two was significant in the later stage. This shows that the time-point MPM group had almost no attention consumption to the monitoring of PM cues in the non-target stage, while the participants invested more attention resources for monitoring in the target stage. During the execution of four PM tasks, evidence of control processing and automatic processing appeared alternately, which was in line with the prediction of dynamic multiprocess theory, that is, individual attention resources were flexibly invested in different stages (the non-target stage was characterized by automatic processing, and the target stage was characterized by control processing), and the PM interference effect also mainly manifested in the later stage of the task. However, the accuracy of ongoing tasks in the time-period MPM group was significantly lower than that of the baseline group during the entire stages, indicating that, although the time clue of the time-period MPM reduced the attention consumption in the early and middle stages, it did not reach the level of automatic processing.

Compared with the time-period MPM, the time information of the time-point MPM is more explicit. So, how does this difference cause individual attention allocation to change? The third purpose of this study was to explore the differences in the processing mechanisms of different types of MPM. The performance of the ongoing task was directly compared between the time-point MPM and time-period MPM, and it was found that they were only different under high cognitive load. In the early and middle stages of the task, the time-point MPM did not have the PM interference effect in the non-target stage where PM cues did not appear (since the accuracy and response time of the ongoing task were not different from the baseline group), but the time-period MPM also had PM interference effect in the non-target stage (since the accuracy of the ongoing task was significantly lower than the baseline group). This indicates that the time-period MPM consumed attention resources under high cognitive load when PM cues had not yet appeared. The results imply that different types of MPM have different target specificity in allocating attention resources under complex background tasks. Due to the apparent time cues, the time-point MPM invested more attention resources for cue monitoring only in the later stage close to the emergence of PM cues. However, the time-period MPM invested attention resources for cue monitoring in the entire stages. However, the attention monitoring in the later stage was still significantly more than that in the early and middle stages. This verifies the results of previous studies, indicating that individuals consume more attention resources under uncertain situations (Rose et al., 2010).

Based on the research of Chen et al. (2010), this study divided MPM into time-point MPM and time-period MPM according to the clarity of time clues and explored the impact of cognitive load on the processing mechanism of different types of MPM under the framework of dynamic multiprocess theory for the first time, which has certain theoretical significance. There were three main findings: (1) the performance of the time-point MPM was better than that of the time-period MPM and EBPM; (2) under the condition of high cognitive load, the attention consumption of the time-point MPM and time-period MPM displayed dynamic changes; and (3) the results of this study support dynamic multiprocess theory. It should be noted that this study had some limitations. For example, the PM cues used in the study were non-significant multiple cues. However, the retention and retrieval of PM intention may reach automatic processing under the condition of significant cues and single cue (Harrison and Einstein, 2010; Wang et al., 2011). In this case, will the attention consumption of the MPM groups still show dynamic changes? Will their PM performance benefit from additional time information? Further examination of these questions is needed in the future. Moreover, the time point for the appearance of PM cues in this study was about 1 min, which was a relatively short time interval. It was difficult for participants to have a large attention fluctuation within such a short time interval. A longer time interval would make the change of attention more sufficient and obvious, which might make the dynamic change of attention more clearly manifested. Future research needs to consider the impact of time interval on attention changes.

## Data Availability Statement

The original contributions presented in the study are included in the article/supplementary material, further inquiries can be directed to the corresponding author.

## Ethics Statement

The studies involving human participants were reviewed and approved by Ethics Committee of Henan University. The patients/participants provided their written informed consent to participate in this study. Informed consent was obtained from all individual participants included in the study.

## Author Contributions

JG and EW contributed to conception and design of the study and organized the database. YG performed the statistical analysis and wrote the first draft of the manuscript. All authors contributed to the article and approved the submitted version.

## Funding

This work was supported by the National Social Science Fund of China (20FJKBL005), Philosophy and Social Sciences Planning Project of Henan (2020BJY010), and Postgraduate Education Innovation Project of Henan University (SYL20060122). Philosophy and Social Science Innovation Team Cultivation Project of Henan University (2019CXTD009).

## Conflict of Interest

The authors declare that the research was conducted in the absence of any commercial or financial relationships that could be construed as a potential conflict of interest.

## Publisher’s Note

All claims expressed in this article are solely those of the authors and do not necessarily represent those of their affiliated organizations, or those of the publisher, the editors and the reviewers. Any product that may be evaluated in this article, or claim that may be made by its manufacturer, is not guaranteed or endorsed by the publisher.
